# The Efficacy of Adjunctive Aids in Periodontal Maintenance Therapy: A Systematic Literature Review and Meta-analysis

**DOI:** 10.3290/j.ohpd.a45406

**Published:** 2020-10-13

**Authors:** Egle Ramanauskaite, Urte Marija Sakalauskaite, Vita Machiulskiene

**Affiliations:** a PhD Student, Clinic of Dental and Oral Pathology, Lithuanian University of Health Sciences, Kaunas, Lithuania. Idea, wrote the manuscript.; b Postgraduate Student in Periodontology, Clinic of Dental and Oral Pathology, Lithuanian University of Health Sciences, Kaunas, Lithuania. Literature search, proofread the manuscript.; c Professor, Clinic of Dental and Oral Pathology, Lithuanian University of Health Sciences, Kaunas, Lithuania. Advisor, proofread the manuscript.

**Keywords:** periodontal maintenance, periodontitis, residual pockets, supportive periodontal treatment

## Abstract

**Purpose:**

To evaluate the efficacy of adjunctive aids to scaling and root planing (SRP) on clinical outcomes in treating periodontal patients included in regular periodontal maintenance programs.

**Materials and Methods:**

The electronic databases MEDLINE (Pubmed), EMBASE, and the Cochrane Central Register of Controlled Trials (CENTRAL) were searched for relevant articles published up to 1st January, 2020. Randomised controlled clinical trials of SRP with or without the use of adjuncts and published in English were included. A meta-analysis using the random-effects model was performed on the selected qualifying articles.

**Results:**

Nineteen studies were included in the systematic review and sixteen in the meta-analysis. The overall effect of adjunctive aids was statistically significant for reduction in probing depth (PD) (0.376 mm, 95% CI [0.144 to 0.609]) and clinical attachment level (CAL) gain (0.207 mm, 95% CI [0.0728 to 0.340]). No statistically significant differences were observed for changes in bleeding on probing (BOP) (p > 0.05). Among the different adjuncts, statistically significant positive effects were demonstrated for adjunctive photodynamic therapy (PD reduction 0.908 mm, 95% CI [0.227 to 1.589] and CAL change (0.457 mm, 95% CI [0.133 to 0.782]) and tetracycline fibers (PD reduction 0.534 mm, 95% CI [0.290 to 0.778] and CAL gain 0.280 mm, 95% CI [0.0391 to 0.521]).

**Conclusions:**

Despite high heterogeneity of the investigated data, based on the findings of a current systematic review, adjunctive aids (in particular, photodynamic therapy and tetracycline fibers) combined with SRP provide statistically significant clinical benefits compared to SRP alone. Due to the large number of included studies with high risk of bias, future studies should be based on adequate methodological procedures to improve the overall quality of reporting and to reduce the risk of bias.

Periodontitis is a chronic multifactorial inflammatory disease that is associated with dysbiotic plaque biofilms and characterised by progressive destruction of the tooth-supporting apparatus.^52^ The main goals of periodontal therapy include arresting the disease progression and establishing healthy, stable, and maintainable periodontal conditions. A successfully treated stable periodontitis patient should exhibit ≤ 4 mm of PD and < 10% BOP.^9^ Nevertheless, periodontal pockets, which are defined as ‘residual,’ often remain after nonsurgical treatment.^22,40^ The presence of residual pockets may jeopardise tooth survival, be a determinant of further disease progression, and may ultimately lead to tooth loss.^22^ It is well established that a residual PD of 5 mm represents a risk factor for further tooth loss.^40^

In order to prevent the rebound of periodontal pathogens in subgingival plaque, repeated instrumentation and mechanical removal of subgingival plaque are essential, including the subgingival debridement of pockets ≥ 4 mm.^1^ Therefore, treated periodontitis patients should receive periodontal maintenance and be closely monitored.^9^

Maintenance after completion of active periodontal therapy includes three basic components: measures taken by the patient (personal oral hygiene, avoidance of environmental risks, management of systemic diseases), preventive procedures carried out by a dental health-care professional (removal of supragingival deposits and polishing, elimination of plaque-retentive factors), and supportive periodontal therapy (interventions addressing the cause and physio-pathological mechanisms of recurrent disease).^44^

Recent studies show that, when managing untreated periodontal disease, the outcomes of periodontal therapy may be enhanced by using additional systemic^12,16^ or local antibiotics,^3,36,55^ as well as antiseptics^49-51^ or nonsurgical lasers.^2,20^ Until now, only limited evidence has demonstrated clinical outcomes following the application of adjunctive aids to SRP when treating patients with recurrent periodontitis.

The aim of this study was to assess existing evidence of the potential clinical benefits of using adjunctive aids to SRP in periodontal maintenance therapy.

## Materials and Methods

This systematic analysis report adhered to the Preferred Reporting Items for Systematic Review and Meta-Analyses (PRISMA) statement.^43^

### Focus Question

The following focus question was developed regarding the population, intervention, comparison, outcome, and study design (PICOS) (Table 1): In patients with recurrent periodontitis, does the use of local antiseptics, antibiotics, or nonsurgical lasers (as adjuncts to SRP) result in greater improvement of PD, CAL, and BOP, compared to SRP alone?

**Table 1:** The focus question development according to PICOS

Component	Description
Population (P)	Systemically healthy patients, older than 18 years, diagnosed with recurrent periodontitis and included in regular periodontal maintenance programs.
Intervention (I)	For the test groups, SRP plus adjunctive aids (locally delivered antiseptics, antibiotics, nonsurgical lasers).
Comparison (C)	SRP alone or with a placebo.
Outcomes (O)	The primary outcome variable was the changes in pocket probing depths (PDs); secondary outcome variables included changes in clinical attachment level (CAL) and/or bleeding on probing (BOP).
Study design (S)	Randomised controlled clinical trials (RCTs) with parallel or split-mouth designs with a minimum duration of 3 months.

### Information Sources

The electronic databases MEDLINE (PubMed), EMBASE, and the Cochrane Central Register of Controlled Trials (CENTRAL) were searched for relevant articles that had been published until January 1, 2020. The search was limited to human studies and those in the English language.

In addition, manual search of the bibliographies of all full-text articles and the following scientific journals was performed: ‘The International Journal of Periodontics and Restorative Dentistry’, ‘Journal of Clinical Periodontology’, ‘The Journal of Periodontology’, and ‘The Journal of Periodontal Research’.

### Search

The following search terms were used: (“chronic periodontitis” [MeSH term] OR “periodontal disease” [MeSH term] OR “periodontitis” [MeSH term] OR “recurrent periodontitis” [MeSH term] OR “refractory periodontitis” [MeSH term] OR “residual pockets” [MeSH term] AND “treatment” [MeSH term] OR “periodontal maintenance care” [MeSH term] OR “ periodontal supportive care” [MeSH term] OR “therapy” [MeSH term] OR “scaling and root planing” [MeSH term] OR “subgingival debridement” [MeSH term] OR “subgingival irrigation” [MeSH term] OR “photodynamic therapy” [MeSH term] OR “antibiotics” [MeSH term] OR “lasers” [MeSH term] OR “antiseptics” [MeSH term]).

### Selection of Studies

During the first literature selection stage, the titles and abstracts of all identified studies were screened for eligibility by two independent reviewers (ER and UMS).

### Inclusion and Exclusion Criteria

The following inclusion criteria were applied:

- Randomised controlled clinical trials (RCTs) comparing the effectiveness of adjunctive therapies to SRP in patients, diagnosed with a recurrent periodontits;
- Patients in included studies must have received an active periodontal treatment first and been involved in regular periodontal maintenance programs;
- Parallel and split-mouth design studies including systemically healthy patients;
- The presence of a control group, receiving subgingival debridement either alone or with a placebo;
- The test group received the same subgingival debridement as a control group, plus the adjunctive aids, applied subgingivally (locally delivered antiseptics, antibiotics, nonsurgical lasers);
- Subgingival debridement carried out by ultrasonics and/or Gracey curettes;
- The study reported on clinical treatment outcomes, including PD and/or CAL and/or BOP changes before and after treatment;
- Follow-up after the intervention no less than 3 months;
- English language.

In the second stage, the full texts of potentially eligible articles were reviewed and evaluated according to the following exclusion criteria: studies including patients with systemic diseases; studies where adjunctive aids were applied before or after periodontal treatment.

Differences between reviewers were solved through discussion until a consensus was reached. All studies excluded at this stage were recorded, as well as the reasons for their exclusion (Table 2). The agreement level between the reviewers regarding study inclusion was expressed by Cohen’s kappa.

**Table 2:** Excluded studies and reasons for exclusion

Author	Reason for exclusion
Cappuyns et al^7^	No SRP in test groups
Carvalho et al^8^	No SRP in test or control groups
Cattabriga M et al^35^	Control group did not receive SRP
Da Cruz Andrade et al^13^	No SRP in test or control groups
Eickholz et al^17^	No SRP in test group
Flemming et al^18^	Local antibiotics applied to periodontal pocket 1 week later after SRP
Garret et al^19^	No SRP in test group
Hagi et al^24^	No SRP in test group
Jansson et al^27^	Metronidazol gel applied to periodontal pockets 3-6 months after SRP
Kileen et al^28^	The same study cohort as in Kileen et al 2018^32^
Kolbe et al^30^	No SRP in test groups
Krohn-Dale et al^31^	No SRP in test group
Kruse et al^32^	No SRP in test group
McColl et al^41^	No SRP in test group,
Mongardini et al^45^	Follow-up 1 week
Muller et al^46^	No SRP in test groups
Petersilka et al^54^	No SRP in test group
Ratka-Krüger et al^56^	No SRP in test group
Rodrigues et al^59^	No SRP in control group
Rudhart et al^60^	No SRP in test group
Rühling et al^61^	No SRP in test group
Tomasi et al^65^	No SRP in test group

SRP: scaling and root planing.

### Data Extraction and Data Items

From the selected articles fulfilling the inclusion criteria, the following data were retrieved to data extraction templates: country, study design, periodontal status of included patients, time of involvement in maintenance programs, number of participants, follow-up time, tested products, and patients’ gender, age, and smoking status (Table 3). The number of patients included in the final analysis, evaluated clinical parameters, treatment protocols in test and control groups, and clinical outcomes are presented in Table 4. The mean values and standard deviations of changes in PD reduction, BOP reduction, and CAL gain following treatment in both the test and control groups were extracted for data analysis and are also presented in Table 4.

**Table 3:** Material and methods of the selected studies: country, study design, periodontal status of included cohorts, number of patients included in the study, follow-up time, patients’ gender, age, smoking status, and tested products

Study	Country	Study design	Study population (diagnosis)	Level of residual/ persistent disease (at the baseline visit of SPT)	Time in periodontal maintenance care	Participants (control/ test) at the beginning of the study	Follow-up	Gender (M/F)	Smokers	Mean age (range)	Product tested
Grzech-Lesniak K et al, 2019^23^	Poland	Parallel RCT	CP	PD ≥ 5 mm at single-rooted teeth	NR	40 20/20	6 months	15M/25F	Excluded	50.3 ± 11.6 (32–79)	PDT
Megally A et al, 2019^42^	Switzerland	Parallel RCT	Study subjects previously treated for periodontal disease, with evidence of persistent periodontal pockets	PD ≥ 5 mm at single-rooted teeth	At least three months after completion of basic periodontal therapy	32 16/16	12 months	21M/11F	Included	61.9 ± 9.3	Amino acid/ hypochlorite gel
Kileen AC et al, 2018^29^	USA	Parallel RCT	Moderate-severe CP	≥ 5 mm posterior interproximal pocket with a history of BOP	≥ 2 years	55 28/27	24 months	38M/17F	Included	67.1 ± 11.4	Minocycline microspheres
Goh EX et al, 2017^21^	Singapore	Split-mouth RCT	CP	At least two residual pockets of ≥ 5 mm in different quadrants, with or without BOP	NR	27	3 months	11M/16F	Included	55.5 ± 7.9 (44–70)	PDT
Corrêa MG et al, 2016^11^	Brazil	Split-mouth RCT	CP	At least two contra- lateral single-rooted teeth with residual PD ≥ 5 mm and BOP	In SPT for three months, after cause-related therapy	20	3 months	55.6%M/44.44F	Excluded	48.1 ± 7.5	PDT
Nguyen NT et al, 2015^48^	USA	Split-mouth RCT	CP	One or more periodontal sites with PD ≥ 5 mm and BOP	NR	22	3 months	13M/9F	Included	61.8 (47–81)	Diode laser
Campos GN et al, 2013^6^	Brazil	Split-mouth RCT	CP	At least two contralateral single-rooted teeth with residual PD ≥ 5 (BOP)	At least 3 months after completion of basic periodontal therapy	15	3 months	55.6M/44.44F	Excluded	48.15 ± 7.53	PDT
Matesanz P et al, 2013^39^	Spain	Parallel-arm RCT	History of periodontal disease as demonstrated by generalised radiographic bone loss	PD ≥ 4 mm, BOP	At least one year in a supportive periodontal therapy	22 12/10	6 months	8M/14 F	Included	50.1 ± 9 (36–71)	1.5% XAN-CHX gel
Slot et al 2012^63^	Holland	Split-mouth RCT	Moderate-severe CP	≥ 1 site per quadrant with PD of ≥ 5 mm and interproximal attachment loss of ≥ 2 mm, presence of BOP, radiographic evidence of bone loss	In regular SPT for > 1 year	32	3 months	14 M/18F	Included	48.7 ± 11.3 (39–65)	Nd: YAG laser
Tonetti M et al, 2012^66^	Switzerland, Belgium, Germany, Greece, Netherlands	Parallel-group, multi-center RCT	Persistent or recurrent moderate-severe periodontitis	≥ 4 teeth with residual PD ≥ 5 mm and positive BOP	In regular SPT ≥ 6 months	202 102/100	12 months	80M/122F	Included	50	14% doxycycline gel
Dannewitz B et al, 2009^14^	Germany	Parallel-arm RCT	Recurrent moderate-severe periodontitis	At least four teeth with residual PPDs of ≥ 5 mm and a positive BOP	Minimum period of two years	39 20/19	12 months	16M/23F	Included	51.5 ± 9	14% doxycycline gel
Lulic et al, 2009^34^	Switzerland	Split-mouth RCT	CP	PD ≥ 5 mm with/without concomitant BOP	Patients in maintenance care for a mean of 11.3 years	10	Six months	3M/7F	Included	54 (40–74)	PDT
Chondros P et al, 2009^10^	Holland	Parallel-arm RCT	CP	At least one site per quadrant with PD ≥ 4 mm with BOP	NR	24 12/12	Six months	10M/14F	Included	49.45 ± 8.62	PDT
Bogren A et al, 2008^4^	Sweden	Parallel-arm RCT	Moderate-advanced CP	Minimum four teeth with PD ≥ 5 mm	In SPT ≥ 1 years	128 65/63	36 months	41.5%M/58.5F	Included	59 (34–82)	8.8% doxycycline gel
Leiknes T et al, 2007^33^	Norway	Split-mouth RCT	NR	PD ≥ 5 mm and BOP	≈2–3 years after primary periodontal treatment	21	Six months	10M/11F	NR	50.3	25% metronidazole gel
Heasman PA et al, 2001^25^	United Kingdom	Split-mouth RCT	Moderate-severe CP	Minimum one pocket/ quadrant with a PD ≥ 5 mm, with persistent BOP	NR	26	Six months	8M/18F	NR	42.6 ± 12.6 (35–59)	CHX chip
Riep B et al, 1999^58^	Germany	Split-mouth RCT	Localised recurrent periodontitis	Non-adjacent sites in different quadrants with PD ≥ 6 mm and BOP	NR	30	Three months	1NR	NR	47	25% metronidazole gel
Wong MY et al, 1998^68^	Taiwan	Split-mouth RCT	Localised recurrent periodontitis	At least two non-adjacent sites with PD 4–8 mm, BOP	NR	30	Six months	19M/11F	Included	42.7	Tetracycline fibers
Newman MG et al, 1994^47^	USA	Multi- center split-mouth RCT	NR	At least two sites in different quadrants with PD of 5–8 mm, BOP	NR	113	Six months	NR	NR	51	Tetracycline fibers

BOP: bleeding on probing; CP: chronic periodontitis; CHX: chlorhexidine; F: female; M: male; NR: not reported; PD: probing depth; PDT: photodynamic therapy; RCT: randomised controlled clinical trial; SPT: supportive periodontal therapy; XAN: xanthan.

**Table 4:** Treatment protocols and changes in PD, CAL, and BOP in test and control groups

Author, year	Number of patients that completed the study (Test/Control)	Treatment protocol in control group	Treatment protocol in test group	Outcomes	Change in PD	Change in CAL	Change in BOP	Comments
Grzech-Lesniak K, 2019^23^	40 20/20	SRP	SRP + PDT (PDT at baseline, 7 and 14 days after baseline)	FMPS (full mouth measurements); BOP, PD, CAL, REC (at the treated sites)	Control: 0.29 ± 0.66 Test: 0.32 ± 0.69	-	Control: 1 ± 6.7 Test: 5.3 ± 6.9	Statistically significantly greater decrease in BOP in the test group (p = 0.007)
Megally A et al, 2019^42^	32 16/16	Ultrasonics	Ultrasonics + hypochlorite/ amino acid gel (repeated at 4 and 8th-month follow-up visits)	PI, PD, BOP, REC (full mouth measurements)	Control: 0.85 ± 1.13 Test: 0.97 ± 1.09	Control: 0.82 ± 1.33 Test: 1.02 ± 1.49	-	NS
Kileen AC et al, 2018^29^	48 25/23	SRP	SRP + Minocycline microspheres (repeated at six- and 12-month follow-up visits)	PD, CAL, BOP (at the treated sites)	Control: 1.1 ± 0.6 Test: 0.8 ± 0.9	Control: 1.0 ± 0.6 Test: 0.8 ± 0.9	-	NS
Goh EX et al, 2017^21^	27	SRP	SRP + PDT (single application at baseline)	PPD, REC, CAL, PI, BOP (at the treated sites)	Control: 0.56 ± 0.15 Test: 0.82 ± 0.18	Control: 0.6 ± 0.22 Test: 0.65 ± 0.25	-	NS
Corrêa MG et al, 2016^11^	15	SRP + photosensitiser, the laser was positioned but not activated	SRP + PDT (single application at baseline)	PGM, RCAL, PPD (at the treated sites)	Control: 1.0 ± 0.8 Test: 2.3 ± 0.8	Control: 0.3 ± 0.7 Test: 1.3 ± 1.6	3 months	Statistically significant changes in PD and CAL in the test group, compared to the control group (p < 0.05)
Nguyen NT et al, 2015^48^	22	SRP	SRP + diode laser (single application at baseline)	PD, BOP, REC, CAL (at the treated sites)	Control: 0.91 ± 0.7 Test: 0.93 ± 0.7	Control: 0.68 ± 1.17 Test: 0.53 ± 1.17	Control: 25 ± 28.14 Test: 28 ± 28.14	NS
Campos GN et al, 2013^6^	13	SRP	SRP + PDT (single application at baseline)	FMPS, FMBS (full mouth measurements) PGM, CAL, PPD, BOP (at the treated sites)	Control: 1.14 ± 1.53 Test: 2.17 ± 0.91	Control: 0.51 ± 0.76 Test: 1.43 ± 1.61		Higher PD reduction and CAL gain observed in PDT + SRP group at three months (p < 0.05)
Matesanz P et al, 2013^39^	21 11/10	SRP + placebo gel	SRP + CHX-XAN gel (single application at baseline)	PII, BOP, PPD, REC, CAL (at the treated sites)	Control: 0.22 ± 0.52 Test: 0.32 ± 0.26	Control: 0.04 ± 0.7 Test: 0.3 ± 0.7	Control: 17 ± 17 Test: 14 ± 19	NS
Slot et al 2012^63^	30	SRP	SRP + Nd: YAG laser (single application at baseline)	PPD, REC, BOP (full mouth measurements)	Control: 0.85 ± 0.45 Test: 0.97 ± 0.58	-	Control: 7 ± 24 Test: 2 ± 21	NS
Tonetti M. et al, 2012^66^	200 100/100	SRP + placebo gel	SRP + 14% doxycycline gel (single application at baseline)	PPD, BOP, PAL, PCR, REC (at the treated sites)	Mean changes experienced for each of the initial PPDs were reported (4, 5, 6, 7, 8 mm, or more), instead of absolute numerical values	Results were expressed as adjusted mean changes in PAL between test and control treatments by baseline pocket depth (4, 5, 6, 7, and 8+ mm) at three-, six-, and 12-month follow-up	BOP was expressed as the OR for treatment difference in the rate of healing of sites, with PPD 5 mm or more, or 4 mm with BOP as a category of non-bleeding sites, with PPD 4 mm or more	NS
Dannewitz B. et al, 2009^14^	34 19/15	Ultrasonic instrumentation	Ultrasonic instrumentation + 14% doxycycline gel (single application at baseline)	FMPS, FMBS (full mouth measurements) BOP, PPD, REC (at the treated sites)	Control: 0.7 ± 1.1 Test: 0.88 ± 1.3	Control: 0.89 ± 2.2 Test: 1.27 ± 2.3	-	NS
Lulic et al, 2009^34^	10	SRP + non-activated laser	SRP + PDT (PDT repeated at one, two, seven, and 14 days)	PII, PPD, CAL, BOP (at the treated sites)	Control: 0.04 ± 0.33 Test: 0.67 ± 0.34	Control: 0.27 ± 0.52 Test: 0.52 ± 0.31	-	Statistically significant changes in PD and CAL, in favour of the test group
Chondros P et al, 2009^10^	24 12/12	Sonic scaler	Sonic scaler + PDT (single application at baseline)	FMPS, FMBS (full mouth measurements) PPD, REC, CAL (at the treated sites)	Control: 0.9 ± 0.8 Test: 0.8 ± 0.5	Control: 0.5 ± 0.6 Test: 0.7 ± 0.7	Control: 1 ± 12.17 Test: 3 ± 10.44	Statistically significant reduction of BOP in favour of the test group
Bogren A et al, 2008^4^	124 64/60	SRP	SRP + doxycycline gel (baseline, one year, and two years)	FMPS, BOP, PD, GM (full mouth measurements)	Control: 1.1 ± 0.8 Test: 1.2 ± 0.55	Control: 0.2 ± 0.6 Test: 0.1 ± 0.97	Control: 18 ± 22.95 Test: 19 ± 26.9	NS
Leiknes T et al, 2007^33^	21	SRP	SRP + 25% metronidazole gel (repeated after one week)	PD, RAL, BOP (at the treated sites)	Control: 1.8 ± 0.5 Test: 1.9 ± 1.06	Control: 1.0 ± 1.65 Test: 1.6 ± 1.41	-	NS
Heasman PA et al, 2001^25^	24	SRP	SRP + CHX chip (single application at baseline)	PI, PPD, BI, CAL (at the treated sites)	Control: 0.45 ± 0.64 Test: 0.78 ± 0.59	Control: 0.15 ± 0.44 Test: 0.43 ± 0.73	Control: 45 ± 13 Test: 78 ± 12	Statistically significantly greater improvements in all clinical parameters in test group
Riep B et al, 1999^58^	29	SRP Test:	SRP + 25% metronidazole gel (5 x during a period of ten days)	PPD, CAL, PI (at the treated sites)	Control: 1.7 ± 0.9 Test: 1.7 ± 0.9	Control: 1.1 ± 0.8 Test: 1.3 ± 0.8	--	NS
Wong MY et al, 1998^68^	30	SRP	SRP + tetracycline fibers (single application at baseline)	PI, GI, BOP, PAL, REC (at the treated sites)	Control: 0.92 ± 1.2 Test: 1.38 ± 1.36	Control: 0.75 ± 1.2 Test: 0.8 ± 1.09	--	NS
Newman MG et al, 1994^47^	105	SRP	SRP + tetracycline fibers (single application at baseline)	REC, CAL, BOP (at the treated sites)	Control: 1.08 ± 1.24 Test: 1.81 ± 1.24	Control: 1.08 ± 1.49 Test: 1.56 ± 1.24	---	Statistically significantly greater improvements in all clinical parameters in the test group

BOP: bleeding on probing; CAL: clinical attachment level; FMBS: full mouth bleeding score; FMPS: full mouth plaque score; GI: gingival index; NS: no statistically significant difference between study groups; PI: plaque index; PCR: The patient’s plaque-control record; PGM: position of the gingival margin; PAL: probing attachment level; PD: probing depth; PDT: photodynamic therapy; RAL: relative attachment level; RCAL: relative clinical-attachment level; REC: recession; SRP: scaling and root planing.

Differences (∆) between baseline-end visits that were not reported were calculated according to the formula: ∆Vary = Var2-Var1 (Var1 and Var2 – mean values before and after treatment). The variance was estimated with the formula: SVar2 = SVar1^2^- SVar2^2^ – (2*r*SVar1*SVar2) (SVar1^2^ and SVar2^2^ – variances of the mean baseline and end values; a correlation (r) of 0.5 was assumed.^38,53^

### Risk of Bias Assessment

The quality of all included studies was assessed during the data-extraction process, which involved an evaluation of the methodological elements that could influence each study’s outcome (Table 5). The Cochrane Collaboration’s two-part tool for assessing the risk of bias was used to assess bias across the studies and to identify papers with intrinsic methodological and design flaws.^26^ The following items were evaluated as posing a low, high, or unclear risk of bias: random sequence generation, allocations concealment, the blinding of participants/personnel, incomplete outcome data, selective reporting outcomes, and other potential risks of bias. The degree of bias was categorised as low risk if all criteria were met, moderate risk when one criterion was missing, and high risk if two or more criteria were missing.

**Table 5:** Assesment of the risk of bias

Author, year	Random sequence generation	Allocation concealment	Blinding	Incomplete outcome data	Selective reporting	Other bias
Grzech-Lesniak K et al, 2019^23^	+	?	-	+	?	+
Megally A et al, 2019^42^	+	+	+	+	+	+
Kileen AC et al, 2018^29^	-	+	-	+	+	+
Goh EX et al, 2017^21^	-	+	?	+	+	+
Corrêa MG et al, 2016^11^	+	?	+	+	+	+
Nguyen NT et al, 2015^48^	+	+	+	+	+	+
Campos GN et al, 2013^6^	+	?	+	+	+	+
Matesanz P et al, 2013^62^	+	+	+	+	+	+
Slot et al 2012^63^	?	+	+	+	+	+
Tonetti M. et al, 2012^66^	+	+	+	+	+	+
Dannewitz B et al, 2009^14^	+	+	+	+	+	+
Lulic et al, 2009^34^	+	+	+	+	?	+
Chondros P et al, 2009^10^	?	+	+	+	+	+
Bogren A et al, 2008^4^	+	?	?	+	+	+
Leiknes T et al, 2007^33^	-	?	+	+	+	+
Heasman PA et al, 2001^25^	-	-	-	+	+	+
Riep B et al, 1999^58^	?	-	?	+	+	+
Wong MY et al, 1998^68^	_	_	_	+	+	+
Newman MG et al, 1994^47^	?	+	+	+	+	+

+ = Low risk; ? = unclear risk; - = high risk

### Data Synthesis

All meta-analyses were performed on randomised controlled clinical trials, reporting the clinical outcomes of recurrent periodontitis treatment utilising different adjunctive aids.

Individual trials were pooled, and the overall rates of probing depth reduction, clinical attachment level gains, bleeding-on-probing reduction, and the 95% confidence intervals (CIs) among the treatment groups were calculated. Fixed or random effects models were used based on the presence or absence of heterogeneity among the included studies. The heterogeneity among the included trials was tested by the heterogeneity test using the Cochran Q statistics. We considered that the random-effects model (the DerSimonian-Laird method)^15^ was more appropriate to use in our case because it accounted for the random variation within the studies and the variation among different studies. Later findings indicated that the fixed-effects model might be invalid. Indeed, the random-effects model tended to give a more conservative estimate (i.e. with a wider confidence interval), but the results from the two models usually agreed well.

## Results

### Study Selection

**Fig 1:**
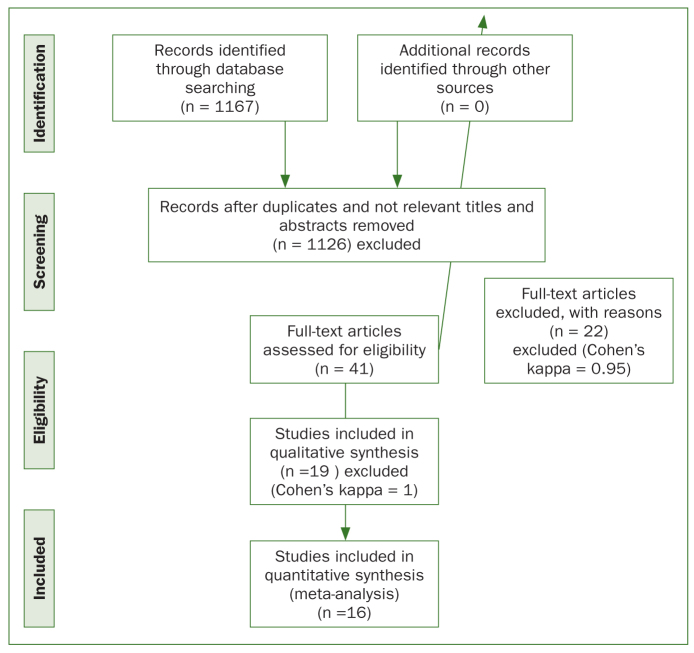
PRISMA flow diagram.

The initial electronic search resulted in the identification of 1167 titles. Following the evaluation of titles and abstracts, 1126 publications were excluded. The remaining 41 full-text articles were evaluated. After applying the inclusion and exclusion criteria, 22 articles were excluded (Cohen’s kappa = 0.95) (Table 2). Finally, 19 RCTs were included in the review (Cohen’s kappa = 1). The study selection process is illustrated in Fig 1.

### Quality Assessment

In terms of the risk of bias for each study, six studies were classified as having a low risk of bias (all domains included),^14,34,39,42,48,66^ four studies had a moderate risk (bias for one key domain),^6,10,11,63^ and nine studies were judged to have a high risk of bias (Table 5).^4,21,23,25,29,33,47,58,68^

### Characteristics of Included Studies

The included studies are outlined in Table 1. Eight studies used a parallel arms design,^4,10,14,23,29,39,42,66^ while the remaining investigations employed a split-mouth design.^6,11, 21,25,33,34,47,48,58,63,68^ Two studies^47,66^ were multi-center randomised RCTs and the remaining investigations were performed in single centers.

With regard to the follow-up period of the included studies, four studies had a follow-up period of three months,^6,11,48,58^ nine studies had a follow-up period of six months,^10,21,23,25,33,39,47,63,68^ four studies had a follow-up period of 12 months,^14,34,42,66^ one study had a follow-up period of 24 months,^29^ and one study had a follow-up period of 36 months.^4^

The present analysis involved a total of 888 patients. In total, 849 (95.6%) patients completed the studies. The mean age of the included patients ranged from 3223 to 82 years^4^ and the ratio of included males and females varied from 0.4034 to 1.90.^42^ Smokers were included in 12 of the studies.^4,10,14,21,29,34,39,42,48,63,66,68^ Smoking habit was an exclusion criterion in 3 investigations,^6,11,23^ whereas patient smoking status was not reported in 4 of the studies.^25,33,47,58^

The time of the patients being involved into regular periodontal maintenance programs ranged from 3 months^6,11,42^ to 11.5 years,^34^ whereas it was not reported in 8 of the studies.^10,21,23,25,33,47,58,68^

With regard to the diagnosis of included patients, seven studies involved patients that had been diagnosed with chronic periodontitis,^6,10,11,21,23,34,48^ four studies reported on patients with moderate-severe or advanced chronic periodontitis,^4,25,29,63^ two studies included patients with a history of periodontal disease,^39,42^ two studies involved patients with recurrent moderate-severe chronic periodontitis,^14,66^ and two studies involved patients that had been diagnosed with a localised recurrent periodontitis.^58,68^ The periodontal diagnosis of the included patients was not reported in two studies.^33,47^

Treatment protocols in the test and control groups are depicted in Table 4. All participants of the included studies had previously received basic periodontal treatment, before randomisation. In all the included studies, subgingival debridement was accomplished by ultrasonics and Gracey curettes, except for three studies, in which subgingival debridement was solely performed by ultrasonics.^10,14,42^ Follow-up visits in the included studies consisted of reinforcement of oral hygiene and supragingival plaque control. Subgingival instrumentation at each follow-up visit was conducted in three studies.^4,21,42^ Additional post-operative rinsing was restricted in all of the included studies, except for three studies, in which patients were instructed to rinse with 0.1-0.12% chlorhexidine.^4,63,68^

### Adjunctive Aids

The included studies were divided into three broad groups, according to their adjunctive aid to SRP:

- Studies that used locally delivered antiseptics and SRP: CHX chips,^25^ CHX-Xanthan gel,^39^ sodium hypochlorite/amino acid gel^42^
- Studies that used locally delivered antibiotics and SRP: minocycline microspheres,^29^ doxycycline gel,^4,14,66^ metronidazole gel,^33,58^ tetracycline fibers^47,68^
- Studies that used nonsurgical lasers and SRP: PDT diode lasers (wavelength: 660–810 nanometers),^6,10,11,21,23,34^ non-PDT diode lasers (wavelength: 808–980 nanometers),^48^ Nd: YAG lasers.^63^

### Synthesis of Results

Meta-analyses were only performed for studies with similar comparisons that reported the same outcome measures.

The first analysis evaluated the overall effect of the adjunctive aids to SRP. Despite high heterogeneity among the included studies (p = 0.000), there were statistically significant differences in favour of the test groups for both changes in PD (weighted mean difference [WMD] = 0.376 mm, 95% CI [0.144 to 0.609] and degrees of freedom [df] = 17; heterogeneity test [Q] = 48.9749; p < 0.0001), as well as changes in CAL (WMD = 0.207 mm, 95% CI [0.0728 to 0.340]; df = 15; Q = 14.3515; p < 0.0001). No statistically significant differences between groups were observed in the overall meta-analysis for changes in BOP (OR = 0.425, 95% CI [-0.174 to 1.024]; df = 6; Q = 41.5024; p = 0.4991).

**Fig 2:**
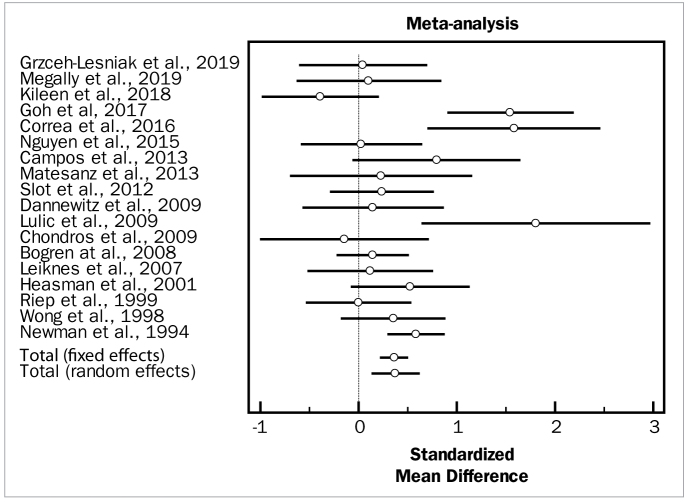
Forest plot of odds ratio (95% CI) for probing depth using adjunctive aids.

**Fig 3:**
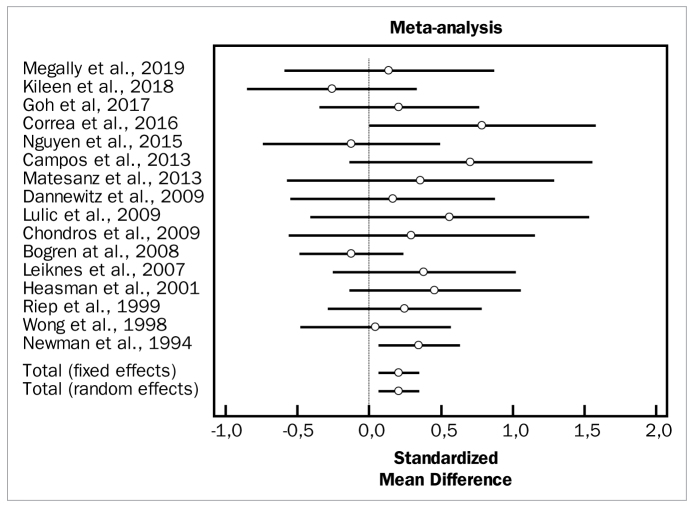
Forest plot of odds ratio (95% CI) for clinical attachment level using adjunctive aids.

**Fig 4:**
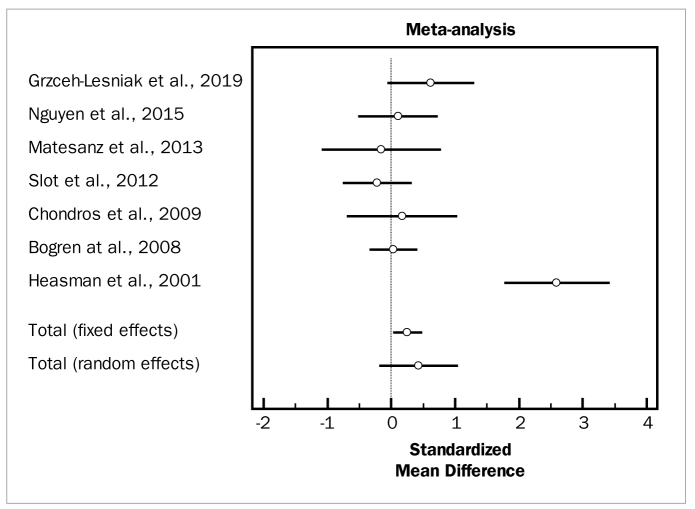
Forest plot of odds ratio (95% CI) for bleeding on probing using adjunctive aids.

Figures 2 to 4 depict forest plots of odds ratios (95% CI) for PD, CAL, and BOP, using adjunctive aids to SRP.

**Table:** 

Study	N1	N2	Total	SMD	95% CI
Grzceh-Lesniak et al, 2019^23^	20	20	40	0.0436	-0.597 to 0.684
Megally et al, 2019^42^	16	16	32	0.105	-0.617 to 0.828
Kileen et al, 2018^29^	23	25	48	-0.389	-0.977 to 0.198
Goh et al, 2017^21^	27	27	54	1,547	0.918 to 2,175
Correa et al, 2016^11^	15	15	30	1,581	0.709 to 2,453
Nguyen et al, 2015^48^	22	22	44	0.0281	-0.580 to 0.637
Campos et al, 2013^6^	13	13	26	0.792	-0.0537 to 1,639
Matesanz et al, 2013^39^	10	11	21	0.230	-0.688 to 1,148
Slot et al, 2012^63^	30	30	60	0.238	-0.281 to 0.757
Dannewitz et al, 2009^14^	15	19	34	0.147	-0.557 to 0.852
Lulic et al, 2009^34^	10	10	20	1,801	0.648 to 2,954
Chondros et al, 2009^10^	12	12	24	-0.145	-0.993 to 0.703
Bogren et al, 2008^4^	60	64	124	0.144	-0.212 to 0.500
Leiknes et al, 2007^33^	21	21	42	0.118	-0.506 to 0.743
Heasman et al, 2001^25^	24	24	48	0.527	-0.0646 to 1,119
Riep et al, 1999^58^	29	29	58	0.000	-0.526 to 0.526
Wong et al, 1998^68^	30	30	60	0.354	-0.167 to 0.875
Newman et al, 1994^47^	105	105	210	0.587	0.309 to 0.865
Total (fixed effects)	482	493	975	0.360	0.232 to 0.489
Total (random effects)	482	493	975	0.376	0.144 to 0.609
Q= 48,9749; df=17; p < 0.0001					

**Table:** 

Study	N1	N2	Total	SMD	95% CI
Megally et al, 2019^42^	16	16	32	0.138	-0.585 to 0.861
Kileen et al, 2018^29^	23	25	48	-0.259	-0.844 to 0.325
Goh et al, 2017^21^	27	27	54	0.209	-0.338 to 0.757
Correa et al, 2016^11^	15	15	30	0.788	0.00722 to 1,569
Nguyen et al, 2015^48^	22	22	44	-0.126	-0.735 to 0.483
Campos et al, 2013^6^	13	13	26	0.708	-0.131 to 1,547
Matesanz et al, 2013^39^	10	11	21	0.357	-0.567 to 1,280
Dannewitz et al, 2009^14^	15	19	34	0.165	-0.540 to 0.870
Lulic et al, 2009^34^	10	10	20	0.559	-0.403 to 1,521
Chondros et al, 2009^10^	12	12	24	0.296	-0.556 to 1,148
Bogren et al, 2008^4^	60	64	124	-0.124	-0.480 to 0.232
Leiknes et al, 2007^33^	21	21	42	0.384	-0.246 to 1,014
Heasman et al, 2001^25^	24	24	48	0.457	-0.132 to 1,046
Riep et al, 1999^58^	29	29	58	0.247	-0.282 to 0.775
Wong et al, 1998^68^	30	30	60	0.0431	-0.474 to 0.560
Newman et al, 1994^47^	105	105	210	0.349	0.0747 to 0.623
Total (fixed effects)	432	443	875	0.207	0.0728 to 0.340
Total (random effects)	432	443	875	0.207	0.0728 to 0.340
Q= 14.3515; df=15; p<0.0001					

**Table:** 

Study	N1	N2	Total	Odds ratio	95% CI
Grzceh-Lesniak et al, 2019^23^	20	20	40	0.620	-0.0373 to 1.277
Nguyen et al, 2015^48^	22	22	44	0.105	-0.504 to 0.714
Matesanz et al, 2013^39^	10	11	21	-0.160	-1.077 to 0.756
Slot et al, 2012^63^	30	30	60	-0.219	-0.737 to 0.300
Chondros et al, 2009^10^	12	12	24	0.170	-0.678 to 1.019
Bogren et al, 2008^4^	60	64	124	0.0399	-0.316 to 0.396
Heasman et al, 2001^25^	24	24	48	2.595	1.790 to 3.399
Total (fixed effects)	178	183	361	0.256	0.0409 to 0.471
Total (random effects)	178	183	361	0.425	-0.174 to 1.024
Q= 41.5024; df=6; p=0.4991					

### Adjunctive Antiseptics and SRP

Three studies,^25,39,42^ which included a total of 77 patients, were included in a meta-analysis for PD and CAL changes. The studies for the investigated parameters did not show statistically significant heterogeneity (p = 0.7456 and p = 0.6752, respectively). For adjunctively applied antiseptics, the meta-analysis found no statistically significant differences in terms of PD reduction (WMD = 0.329 mm, 95% CI [-0.0702 to 0.340]; df = 2; Q = 0.9054; p = 0.6359) or CAL gain (WMD = 0.333 mm, 95% CI [-0.0651 to 0.732]; df = 2; Q = 0.4854; p = 0.7854).

For changes in BOP, two studies^25,39^ with a total of 45 patients were included. A meta-analysis did not indicate a statistically significant reduction in BOP scores for adjunctively applied antiseptics (OR = -1.223, 95% CI [-3.972 to 1.526]; df = 1; Q = 21.5978; p = 0.5268). The included studies demonstrated high heterogeneity (p = 0.000).

**Fig 5:**
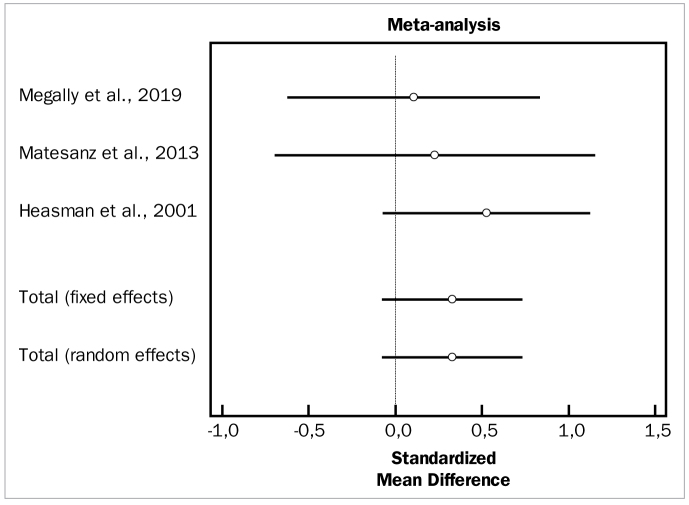
Forest plot of odds ratio (95% CI) for probing depth reduction using adjunctive antiseptics.

**Fig 6:**
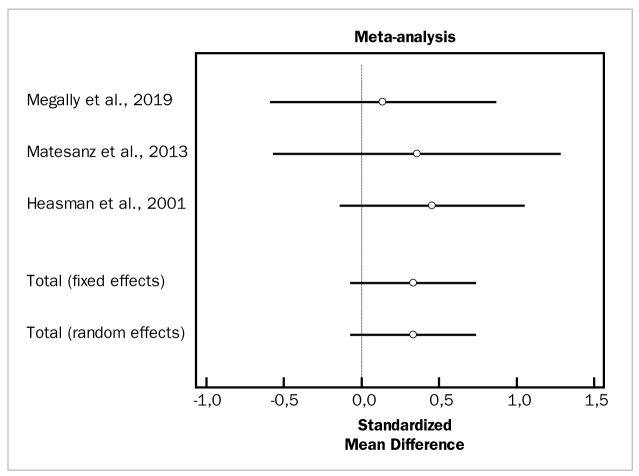
Forest plot of odds ratio (95% CI) for clinical attachment level gain using adjunctive antiseptics.

**Fig 7:**
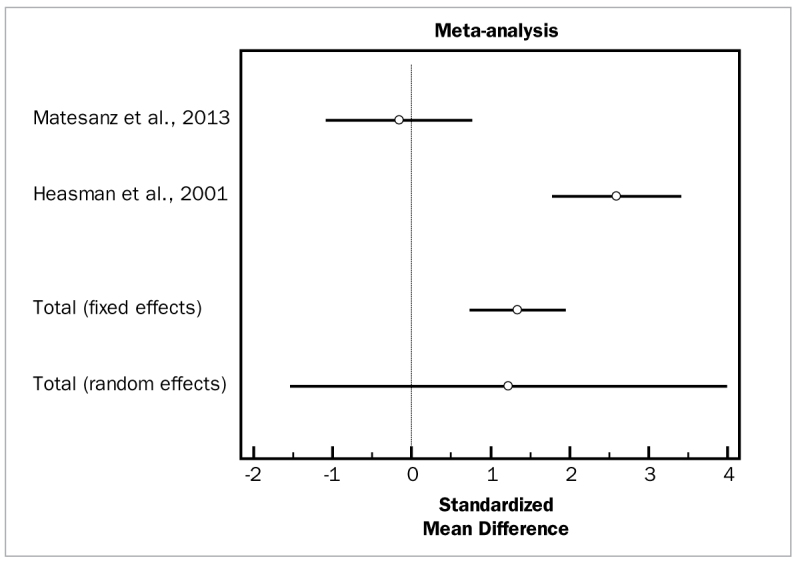
Forest plot of odds ratio (95% CI) for bleeding on probing reduction using adjunctive sustained-release vehicle antiseptics.

Figures 5 to 7 present forest plots of odds ratios (95% CI) for PD, CAL, and BOP, using adjunctive antiseptics for SRP.

**Table:** 

Study	N1	N2	Total	SMD	95% CI
Megally et al, 2019^42^	16	16	32	0.105	-0.617 to 0.828
Matesanz et al, 2013^39^	10	11	21	0.230	-0.688 to 1.148
Heasman et al, 2001^25^	24	24	48	0.527	-0.0646 to 1.119
Total (fixed effects)	50	51	101	0.329	-0.0702 to 0.728
Total (random effects)	50	51	101	0.329	-0.0702 to 0.728
Q= 0.9054; df=2; p=0.6359					

**Table:** 

Study	N1	N2	Total	SMD	95% CI
Megally et al, 2019^42^	16	16	32	0.138	-0.585 to 0.861
Matesanz et al, 2013^39^	10	11	21	0.357	-0.567 to 1.280
Heasman et al, 2001^25^	24	24	48	0.457	-0.132 to 1.046
Total (fixed effects)	50	51	101	0.333	-0.0651 to 0.732
Total (random effects)	50	51	101	0.333	-0.0651 to 0.732
Q= 0.4854; df=2; p=0.7845					

**Table:** 

Study	N1	N2	Total	Odds ratio	95% CI
Matesanz et al, 2013^39^	11	10	21	0.160	-0.756 to 1.077
Heasman et al, 2001^25^	24	24	48	-2.595	-3.399 to -1.790
Total (fixed effects)	35	34	69	-1.342	-1.932 to -0.753
Total (random effects)	35	34	69	-1.223	-3.972 to 1.526
Q= 21.5978; df=1; p=0.5268					

### Adjunctive Locally Delivered Antibiotics and SRP

The overall meta-analysis of adjunctive antibiotics to SRP for PD and CAL changes included seven studies, which included a total of 391 patients.^4,14,29,33,47,58,68^ While the studies for PD demonstrated high heterogeneity (p = 0.000), the same studies in the meta-analysis for CAL did not demonstrate statistically significant heterogeneity (p = 0.4323). The results indicate that, compared to SRP alone, adjunctive locally delivered antibiotics did not improve PD (WMD = 0.185 mm, 95% CI [-0.0687 to 0.438]; df = 6; Q = 12.1507; p = 0.0587) or CAL values (WMD = 0.145 mm, 95% CI [-0.0197 to 0.309]; df = 6; Q = 7.2233 p = 0.3007).

**Fig 8:**
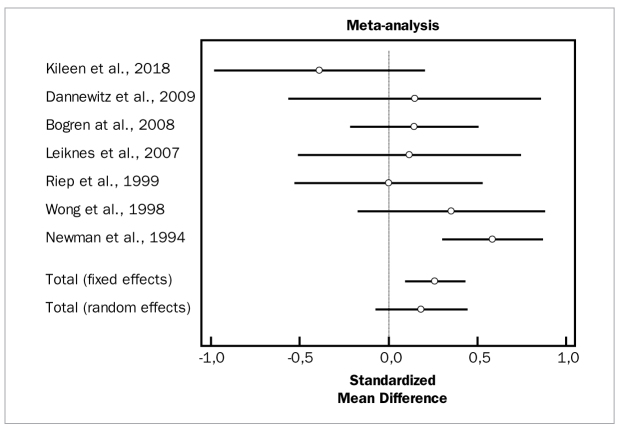
Forest plot of odds ratio (95% CI) for probing depth reduction using adjunctive locally delivered antibiotics.

**Fig 9:**
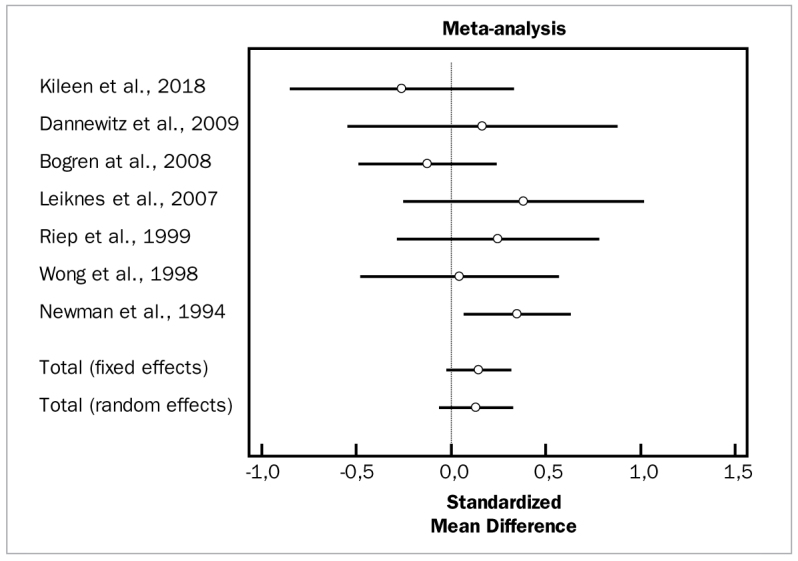
Forest plot of odds ratio (95% CI) for clinical attachment level gain using adjunctive locally delivered antibiotics.

Figures 8 and 9 present forest plots of odds ratios (95% CI) for PD and CAL, using adjunctive antibiotics for SRP.

**Table:** 

Study	N1	N2	Total	SMD	95% CI
Kileen et al, 2018^29^	23	25	48	-0.389	-0.977 to 0.198
Dannewitz et al, 2009^14^	15	19	34	0.147	-0.557 to 0.852
Bogren et al, 2008^4^	60	64	124	0.144	-0.212 to 0.500
Leiknes et al, 2007^33^	21	21	42	0.118	-0.506 to 0.743
Riep et al, 1999^58^	29	29	58	0.000	-0.526 to 0.526
Wong et al, 1998^68^	30	30	60	0.354	-0.167 to 0.875
Newman et al, 1994^47^	105	105	210	0.587	0.309 to 0.865
Total (fixed effects)	283	293	576	0.263	0.0975 to 0.428
Total (random effects)	283	293	576	0.185	-0.0678 to 0.438
Q= 12.1507; df=6; p=0.0587					

**Table:** 

Study	N1	N2	Total	SMD	95% CI
Kileen et al, 2018^29^	23	25	48	-0.259	-0.844 to 0.325
Dannewitz et al, 2009^14^	15	19	34	0.165	-0.540 to 0.870
Bogren et al, 2008^4^	60	64	124	-0.124	-0.480 to 0.232
Leiknes et al, 2007^33^	21	21	42	0.384	-0.246 to 1.014
Riep et al, 1999^58^	29	29	58	0.247	-0.282 to 0.775
Wong et al, 1998^68^	30	30	60	0.0431	-0.474 to 0.560
Newman et al, 1994^47^	105	105	210	0.349	0.0747 to 0.623
Total (fixed effects)	283	293	576	0.145	-0.0197 to 0.309
Total (random effects)	283	293	576	0.132	-0.0563 to 0.320
Q= 7.2233; df=6; p=0.3007					

#### Doxycycline

Two studies evaluated the clinical efficacy of doxycycline for PD and CAL changes.^4,14^ For adjunctively added doxycycline, these studies demonstrated high heterogeneity (p = 0.000) and no statistically significant differences in PD reduction (WMD = 0.145 mm, 95% CI [-0.171 to 0.460; df = 1; Q = 0.0008303; p = 0.9927) or CAL gain (WMD = -0.0626 mm, 95% CI [-0.378 to 0.253]; df = 6; Q = 0.5508; p = 0.4580).

**Fig 10:**
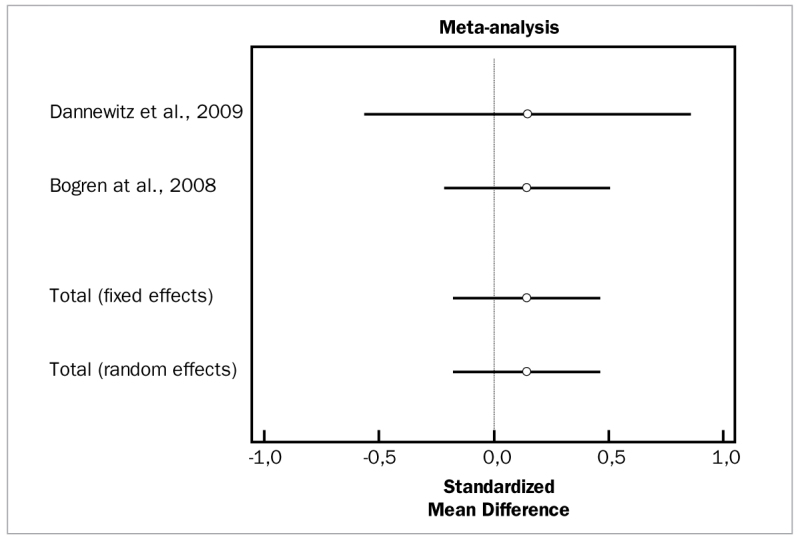
Forest plot of odds ratio (95% CI) for pocket depth reduction using adjunctive doxycycline.

**Fig 11:**
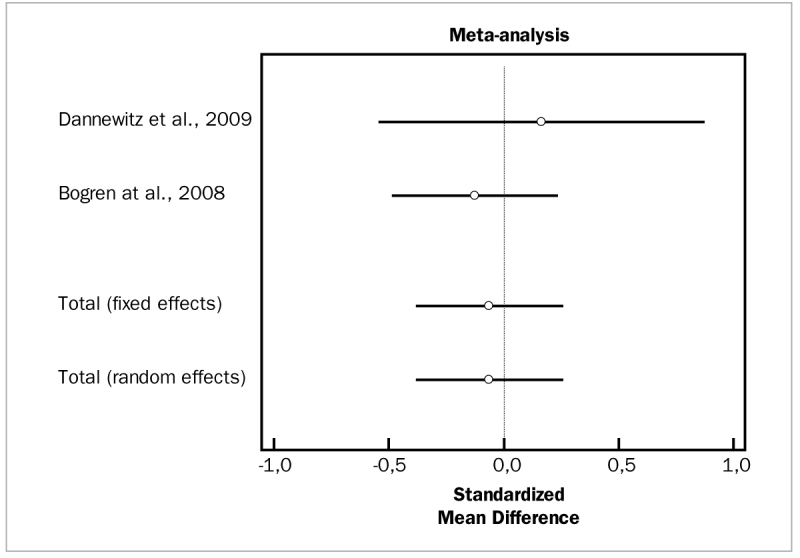
Forest plot of odds ratio (95% CI) for clinical attachment level gain using adjunctive doxycycline.

Figures 10 and 11 illustrate forest plots of odds ratios (95% CI) for PD and CAL, using adjunctive doxycycline for SRP.

**Table:** 

Study	N1	N2	Total	SMD	95% CI
Dannewitz et al, 2009^14^	15	19	34	0.147	-0.557 to 0.852
Bogren et al, 2008^4^	60	64	124	0.144	-0.212 to 0.500
Total (fixed effects)	75	83	158	0.145	-0.171 to 0.460
Total (random effects)	75	83	158	0.145	-0.171 to 0.460
Q= 0.0008303; df=1; p=0.9927					

**Table:** 

Study	N1	N2	Total	SMD	95% CI
Dannewitz et al, 2009^14^	15	19	34	0.165	-0.540 to 0.870
Bogren et al, 2008^4^	60	64	124	-0.124	-0.480 to 0.232
Total (fixed effects)	75	83	158	-0.0626	-0.378 to 0.253
Total (random effects)	75	83	158	-0.0626	-0.378 to 0.253
Q= 0.5508; df=1; p=0.4580					

#### Metronidazole

Two studies evaluated the effects of metronidazole.^33,58^ Data was available for PD and CAL changes. These studies demonstrated high heterogeneity (p = 0.000) and, for the adjunctive metronidazole, no adjunctive effects in terms of PD reduction (WMD = 0.0497 mm, 95% CI [-0.347 to 0.447; df = 1; Q = 0.08526; p = 0.7730) or CAL gain (WMD = 0.304 mm, 95% CI [-0.0958 to 0.703; df = 1; Q = 0.1125; p = 0.7373).

**Fig 12:**
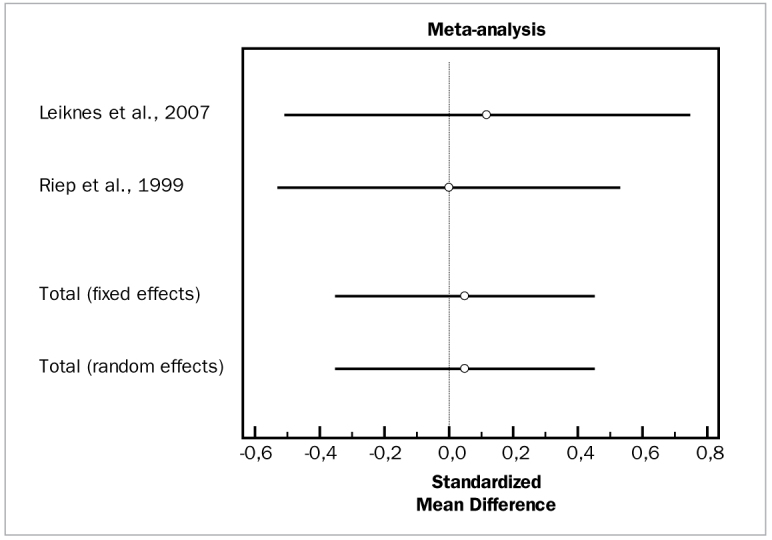
Forest plot of odds ratio (95% CI) for probing depth reduction using adjunctive metronidazole.

**Fig 13:**
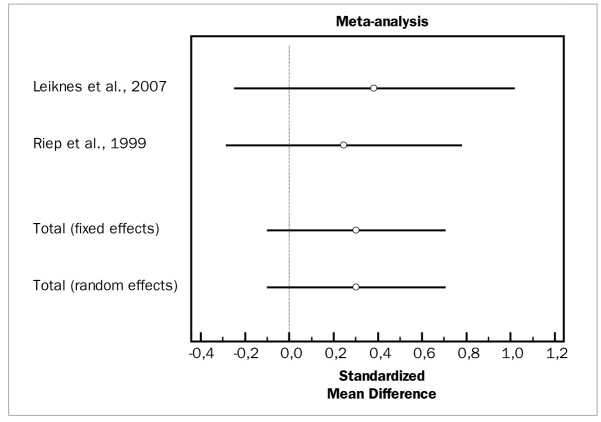
Forest plot of odds ratio (95% CI) for clinical attachment level gain using adjunctive metronidazole.

Figures 12 and 13 present forest plots of odds ratios (95% CI) for PD and CAL, using adjunctive metronidazole for SRP.

**Table:** 

Study	N1	N2	Total	SMD	95% CI
Leiknes et al, 2007^33^	21	21	42	0.118	-0.506 to 0.743
Riep et al, 1999^58^	29	29	58	0.000	-0.526 to 0.526
Total (fixe d effects)	50	50	100	0.0497	-0.347 to 0.447
Total (random effects)	50	50	100	0.0497	-0.347 to 0.447
Q= 0.08526; df=1; p=0.7703					

**Table:** 

Study	N1	N2	Total	SMD	95% CI
Leiknes et al, 2007^33^	21	21	42	0.384	-0.246 to 1.014
Riep et al, 1999^58^	29	29	58	0.247	-0.282 to 0.775
Total (fixed effects)	50	50	100	0.304	-0.0958 to 0.703
Total (random effects)	50	50	100	0.304	-0.0958 to 0.703
Q= 0.1125; df=1; p=0.7373					

#### Tetracycline fibers

Two studies evaluated the adjunctive efficacy of tetracycline fibers, both of which were included in meta-analysis for PD and CAL changes.^47,68^ The studies for the aforementioned parameters did not demonstrate heterogeneity (p = 0.4322 and p = 0.2970, respectively) and the fixed effect model indicated statistically significant PD reductions in the test group (WMD = 0.534 mm, 95% CI [0.290] to 0.778; df = 1; Q = 0.6170; p = 0.0001). Similarly, for changes in CAL, these studies reported statistically significant changes for adjunctive tetracycline fibers (WMD = 0.280 mm, 95% CI [0.0391 to 0.521; df = 1; Q = 1.0875; p = 0.0001).

**Fig 14:**
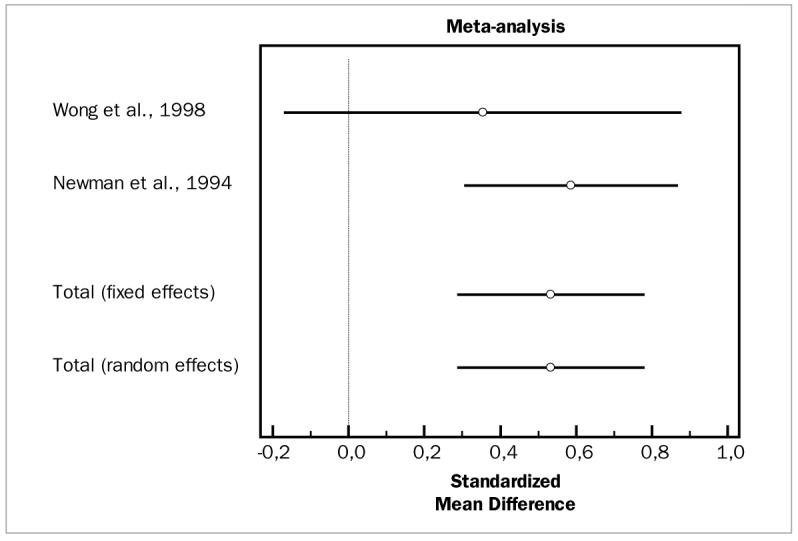
Forest plot of odds ratio (95% CI) for probing depth reduction using adjunctive tetracycline fibers.

**Fig 15:**
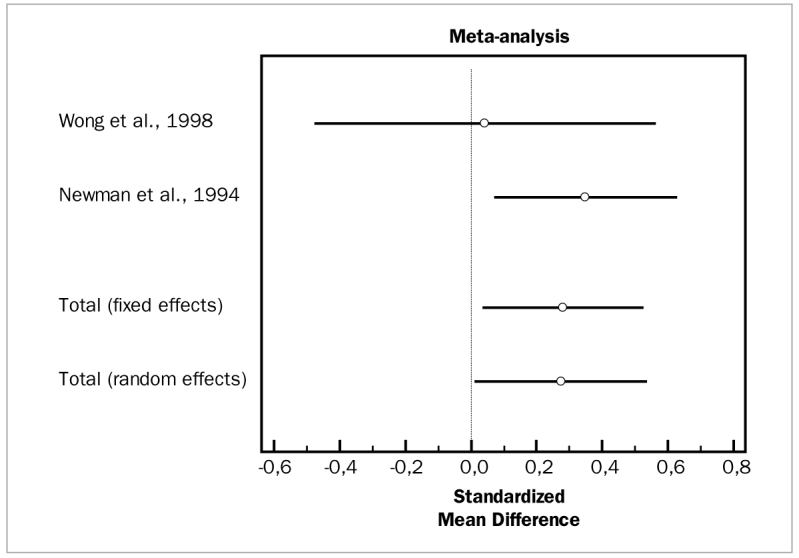
Forest plot of odds ratio (95% CI) for clinical attachment level gain using adjunctive tetracycline fibers.

Figures 14 and 15 illustrate forest plots of odds ratios (95% CI) for PD and CAL, using adjunctive tetracycline fibers for SRP.

**Table:** 

Study	N1	N2	Total	SMD	95% CI
Dannewitz et al, 2009^14^	15	19	34	0.147	-0.557 to 0.852
Bogren et al, 2008^4^	60	64	124	0.144	-0.212 to 0.500
Total (fixed effects)	75	83	158	0.145	-0.171 to 0.460
Total (random effects)	75	83	158	0.145	-0.171 to 0.460
Q= 0.0008303; df=1; p=0.9927					

**Table:** 

Study	N1	N2	Total	SMD	95% CI
Wong et al, 1998^68^	30	30	60	0.354	-0.167 to 0.875
Newman et al, 1994^47^	105	105	210	0.587	0.309 to 0.865
Total (fixed effects)	135	135	270	0.534	0.290 to 0.778
Total (random effects)	135	135	270	0.534	0.290 to 0.778
Q= 0.6170; df=1; p=0.0001					

### Adjunctive Photodynamic Therapy and SRP

For meta-analysis evaluating the effectiveness of adjunctive photodynamic therapy, in terms of PD and CAL changes, six studies were included which involved a total of 129 patients.^6,10,11,21,23,34^ For the meta-analysis of PD changes, the included studies demonstrated high heterogeneity (p = 0.000) and highlighted a statistically significant PD reduction (WMD = 0.908 mm, 95% CI [0.227 to 1.589]; df = 5; Q = 23.2452; p = 0.0003) for the adjunctive use of PDT. In terms of CAL changes, these same studies in the meta-analysis did not demonstrate heterogeneity (p = 0.7232) and revealed a statistically significant CAL change (WMD = 0.457 mm, 95% CI [0.133 to 0.782]; df = 4; Q = 2.1611; p = 0.0001) in favor of the test group.

Based on two studies with a total of 64 patients,^10,23^ the adjunctive application of photodynamic therapy did not statistically significantly reduce BOP scores, compared to SRP alone (OR = 0.446 mm, 95% CI [-0.0621 to 0.954]; df = 1; Q = 0.7406; p = 0.3895). These studies did not demonstrate high heterogeneity (p = 0.642).

**Fig 16:**
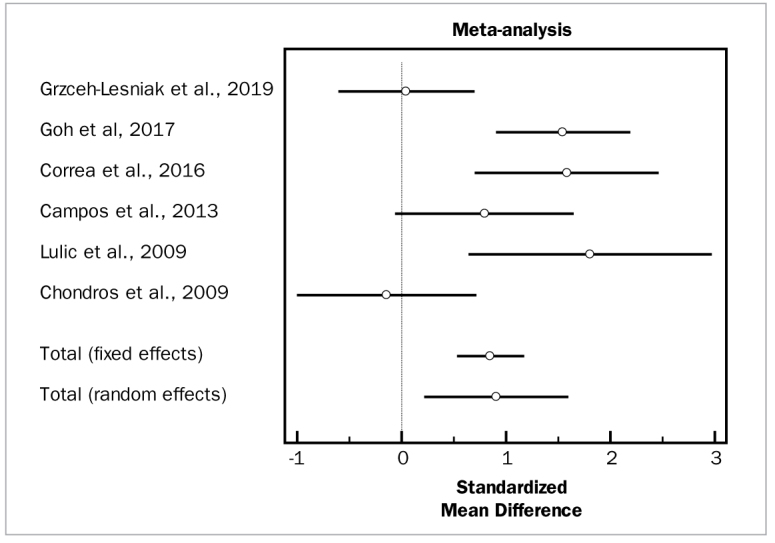
Forest plot of odds ratio (95% CI) for probing depth reduction using photodynamic therapy.

**Fig 17:**
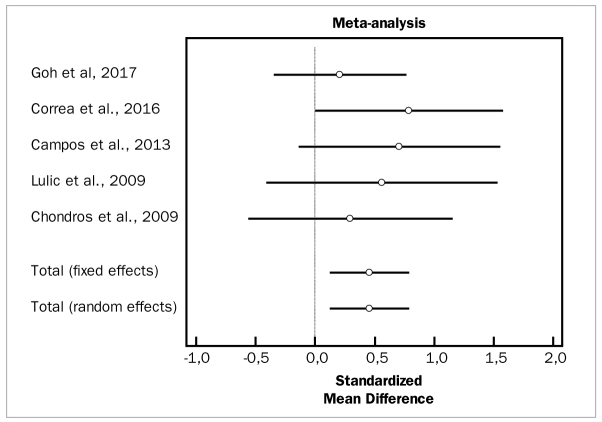
Forest plot of odds ratio (95% CI) for clinical attachment level gain using photodynamic therapy.

**Fig 18:**
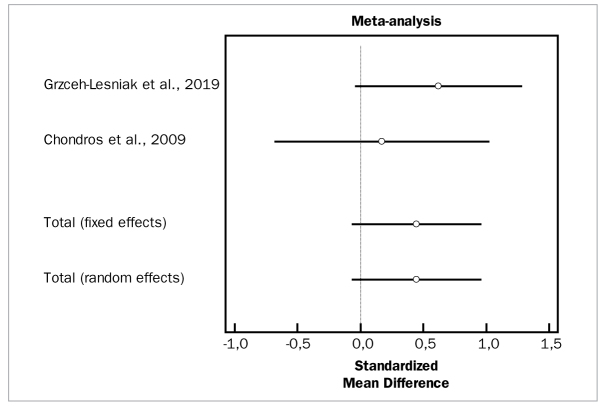
Forest plot of odds ratio (95% CI) for bleeding on probing reduction using photodynamic therapy.

Figures 16, 17, and 18 present forest plots of odds ratios (95% CI) for PD, CAL, and BOP, using adjunctive antiseptics to SRP.

**Table:** 

Study	N1	N2	Total	SMD	95% CI
Grzceh-Lesniak et al, 2019^23^	20	20	40	0.0436	-0.597 to 0.684
Goh et al, 2017^21^	27	27	54	1.547	0.918 to 2.175
Correa et al, 2016^11^	15	15	30	1.581	0.709 to 2.453
Campos et al, 2013^6^	13	13	26	0.792	-0.0537 to 1.639
Lulic et al, 2009^34^	10	10	20	1.801	0.648 to 2.954
Chondros et al, 2009^10^	12	12	24	-0.145	-0.993 to 0.703
Total (fixed effects)	97	97	194	0.849	0.541 to 1.157
Total (random effects)	97	97	194	0.908	0.227 to 1.589
Q= 23.3452; df=5; p=0.0003					

**Table:** 

Study	N1	N2	Total	SMD	95% CI
Goh et al, 2017^21^	27	27	54	0.209	-0.338 to 0.757
Correa et al, 2016^11^	15	15	30	0.788	0.00722 to 1.569
Campos et al, 2013^6^	13	13	26	0.708	-0.131 to 1.547
Lulic et al, 2009^34^	10	10	20	0.559	-0.403 to 1.521
Chondros et al, 2009^10^	12	12	24	0.296	-0.556 to 1.148
Total (fixed effects)	77	77	154	0.457	0.133 to 0.782
Total (random effects)	77	77	154	0.457	0.133 to 0.782
Q= 2.1611; df=4; p=0.0001					

**Table:** 

Study	N1	N2	Total	Odds ratio	95% CI
Grzceh-Lesniak et al, 2019^23^	20	20	40	0.620	-0.0373 to 1.277
Chondros et al, 2009^10^	12	12	24	0.170	-0.678 to 1.019
Total (fixed effects)	32	32	64	0.446	-0.0621 to 0.954
Total (random effects)	32	32	64	0.446	-0.0621 to 0.954
Q= 0.7406; df=1; p=0.3895					

## Discussion

The present study aimed to investigate the potential beneficial effects of adjunctive aids to SRP for treating patients with recurrent periodontitis and enrolled in regular periodontal maintenance programs.

A meta-analysis was based on data extracted from 16 RCTs.^4,6,10,11,14,21,25,28,33,34,39,42,47,48,58,68^ According to our findings, the overall meta-analysis, combining all adjunctive aids, demonstrated statistically significant PD reductions and CAL gains (0.376 and 0.207 mm, respectively; p = 0.000), when compared with the control groups. However, no statistically significant changes were observed in BOP values (p > 0.05). These findings are in agreement with previously published systematic reviews, focusing on the efficacy of adjunctive aids mostly for non-treated periodontal disease and reporting similar changes, ranging between 0.2 and 0.6 mm.^5,38,64^

When the studies were analysed, depending on the adjunctive aid used, the effect was different among the tested products. The greatest PD reduction and CAL gain was observed for adjunctive photodynamic therapy (0.908 and 0.457 mm, respectively; p = 0.000), followed by the adjunctive application of tetracycline fibers (0.534 and 0.280 mm, respectively; p = 0.000). However, despite the beneficial effects of adjunctive tetracycline fibers, the overall meta-analysis of adjunctive local antibiotics did not reveal statistically significant advantages in any of the investigated clinical parameters (p > 0.05). Moreover, the application of antiseptics did not give any additional clinical effect to SRP alone (p > 0.05).

As mentioned above, these results are in line with previous systematic reviews. In particular, Matesanz et al^38^ and Bonito et al^5^ reported statistically significant efficacy in PD reduction for tetracycline fibers (0.727 and 0.47 mm, respectively), whereas Smiley et al^64^ judged photodynamic therapy with a diode laser to be a beneficial adjunct for CAL improvements. Moreover, a meta-analysis of 14 RCTs found statistically significant differences in PD reduction (0.19 mm; p = 0.002) and CAL gain (0.37 mm; p < 0.0001) for adjunctive photodynamic therapy.^62^ However, these comparisons should be considered with caution, as these reviews included patients mostly with untreated periodontal disease.

Literature on the adjunctive aids to SRP, focusing on treating patients with recurrent periodontitis and enrolled in a regular supportive periodontal program (SPT), is scarce. We identified two recent systematic reviews regarding this topic.^37,67^ In the first of them, Trombelli et al^67^ aimed to investigate the efficacy of alternative or additional methods for professional mechanical plaque removal on progression of attachment loss during SPT in periodontitis patients. The review was based on three studies, in which patients in control groups received conventional ultrasonic and hand (curettes) instrumentation, whereas patients in test groups were referred to one of the following treatments: Er:YAG laser, PDT or sub-antimicrobial dose of doxycycline (SDD).^8,31,57^ The pooled data indicated no statistically significant effect of the adjunctive/alternative regimens on CAL change, compared to conventional mechanical instrumentation. The aim of our review was to investigate whether the additional aids, combined with SRP, would enhance clinical periodontal parameters, compared to SRP alone; therefore, two of the studies included in the aforementioned review were excluded from our investigation.^8,31^ The main reason for exclusion was that mechanical plaque removal was not performed in the test groups (Table 2).

Another review investigated the effects of different SPT approaches in adults previously treated for periodontitis.^37^ It included four studies, three of which were included in our review.^29,34,66^ Due to an inadequate number of included studies, the authors were unable to perform meta-analysis and investigate the effect of different variables on clinical outcomes. However, it was concluded that adjunctive treatments may not provide additional benefits, compared to mechanical debridement alone. It should be mentioned that only studies with a follow-up of no less than 12 months were included. Furthermore, RCTs with a split-mouth design were an exclusion criterion in a later study.^37^ Nevertheless, we included the studies with a minimum follow-up of three months and RCTs with a split-mouth design. To justify our inclusion criteria, we presumed that the included patients had already undergone basic periodontal treatment and were involved in regular maintenance programs, and so could have been judged as being compliant and periodontally stable.

The studies identified by the systematic search and included in a current review showed great heterogeneity; therefore, the results must be interpreted with caution. Factors that impact this might include differences in the studied populations, location of periodontal pockets, different formulations, concentrations and parameters of investigated adjunct aids. It should be mentioned that the mode of application of the adjunctive aids also differed among the included studies. In particular, in 12 of the included studies,^6,10,11,14,21,25,38,47,48,63,66,68^ the aids were applied adjunctively to SRP only at the baseline of a study visit, whereas in the remainder of the studies they were applied continuously throughout the study period at different time intervals. The intervals of supportive visits also differed among the studies. As well as the protocols, in three of the studies,^4,21,42^ subgingival debridement was carried out at each follow-up visit, whereas in the other investigations supragingival cleaning or oral hygiene reinforcement were conducted. Moreover, twelve^4,10,14,21,29,34,38,42,48,64,66,68^ out of 19 of the included studies involved patients who smoked. It is a well-established fact that smoking affects periodontal treatment outcomes negatively and is associated with the recurrence of periodontitis during periodontal maintenance, so the results of these studies should be interpreted accordingly.

Only six^14,34,38,42,48,66^ out of 19 studies had a low risk of bias, which included a relatively small number of patients. Other studies were evaluated as having a moderate (n = 4) or high (n = 9) risk of bias. These aspects are important for detecting methodological weaknesses in the included studies that might alter therapy outcomes. According to the results of a bias risk assessment, allocation concealment and the blinding of participants and personnel appeared to be the most critical domains.

Due to the overall high heterogeneity and risk of bias among the studies, future research should be based on adequate methodological procedures to improve the overall quality of the reporting and to reduce risk of bias.

## Conclusions

Despite high heterogeneity of the investigated data, based on the findings of a current systematic review, adjunctive aids (in particular, photodynamic therapy and tetracycline fibers) combined with SRP provide statistically significant clinical benefits compared to SRP alone. Due to the large number of the included studies with high risk of bias, future studies should be based on adequate methodological procedures to improve the overall quality of the reporting and to reduce the risk of bias.
